# Supplementary material for: Philanthropy, audit firms culture and auditor independence
Source: PLoS One. 2022 Nov 10;17(11):e0277567. doi: 10.1371/journal.pone.0277567 (PMC9648830; doi:10.1371/journal.pone.0277567)
Supplement: Attachment — Submitted filename: Response to Reviewers.docx [file pone.0277567.s002.docx]

Dear editor,

Our most sincere thanks to you, Reviewer 1 for the very thoughtful review and helpful comments, and suggestions on our writing in this round.

We have also revised the writing details and formatting of the article based on the reviewers' suggestions, which improved the quality of the article. With your thoughtful comments, our latest revision has improved a lot.

Below, we provide the original comments from Reviewer 1 in boldface and our responses to each item in regular font. We do this point by point.

**Response to Reviewer 1	( PONE-D-22-12398R1)**

**Reviewer #1: The manuscript's research question appealed to me. It has a well-defined research issue, appropriate methods, and innovative findings. The authors have made some changes based on previous comments. Nevertheless, authors could improve the paper further by rearrange the format of the paper to fit with the PLOS One’s formatting guidelines such as title page. There is still minor typo, missing comma and missing space e.g. abstract “Using 5246 audits in the Chinese market between 2010 and 2012,” page 21 “on decisions made in the audit scenario[61].” Or “accruals and audit fees, than when there are none. [61].”, table 1 “ Financial companies” (i.e. you probably want to write as Less: Financial companies”. ), equation 3 “where ACCRjt is total accruals; TAjt is total asset in year t-1;”.**

Thanks for your helpful suggestions!

I correct the details of the minor typo one by one.

(1) According to the PLOS ONE’s formatting guideline, we have further modified the format of the article. It looks better now!

(2) According to the details of the article pointed out by the reviewer, we checked again and revised one by one. For example, Using 5,246 audits in the Chinese market between 2010 and 2012; Less: Financial companies. And we have added all the missing Spaces in the article.
